# Interaction between RNA helicase ROOT INITIATION DEFECTIVE 1 and GAMETOPHYTIC FACTOR 1 is involved in female gametophyte development in Arabidopsis

**DOI:** 10.1093/jxb/erw341

**Published:** 2016-09-28

**Authors:** Dong Zi Zhu, Xue Fang Zhao, Chang Zhen Liu, Fang Fang Ma, Fang Wang, Xin-Qi Gao, Xian Sheng Zhang

**Affiliations:** State Key Laboratory of Crop Biology, College of Life Sciences, Shandong Agricultural University, Taian 271018, China

**Keywords:** Arabidopsis, development, female gametophyte, GFA1, RID1, RNA splicing, U5 snRNP.

## Abstract

ROOT INITIATION DEFECTIVE 1 (RID1) is an Arabidopsis DEAH/RHA RNA helicase. It functions in hypocotyl de-differentiation, *de novo* meristem formation, and cell specification of the mature female gametophyte (FG). However, it is unclear how RID1 regulates FG development. In this study, we observed that mutations to RID1 disrupted the developmental synchrony and retarded the progression of FG development. RID1 exhibited RNA helicase activity, with a preference for unwinding double-stranded RNA in the 3′ to 5′ direction. Furthermore, we found that RID1 interacts with GAMETOPHYTIC FACTOR 1 (GFA1), which is an integral protein of the spliceosome component U5 small nuclear ribonucleoprotein (snRNP) particle. Substitution of specific RID1 amino acids (Y266F and T267I) inhibited the interaction with GFA1. In addition, the mutated RID1 could not complement the seed-abortion phenotype of the *rid1* mutant. The *rid1* and *gfa1* mutants exhibited similar abnormalities in pre-mRNA splicing and down-regulated expression of some genes involved in FG development. Our results suggest that an interaction between RID1 and the U5 snRNP complex regulates essential pre-mRNA splicing of the genes required for FG development. This study provides new information regarding the mechanism underlying the FG developmental process.

## Highlight

RID1 interacts with GFA1 to direct and regulate the splicing and expression of the genes required for female gametophyte development in Arabidopsis.

## Introduction

Angiosperms have evolved to carry the female gametophyte (FG; embryo sac) and male gametophyte (MG; pollen) for sexual reproduction. The MG most commonly consists of two sperm cells and a vegetative cell that produces a pollen tube to deliver the sperm cells to the FG, which is embedded within the ovary (Drews and Koltunow, 2011). The FG is typically composed of seven cells, including one egg cell, two synergids, one central cell, and three antipodal cells. The FG develops from a megaspore mother cell that generates four megaspores through meiosis. The functional megaspore near the chalazal end then undergoes three rounds of mitosis and subsequent cellularization to create the FG. The development of the FG has been extensively studied because of its importance in sexual reproduction (Sundaresan and Alandete-Saez, 2010; Yang *et al.*, 2010). However, some biological processes during FG development have not been fully characterized, such as nuclear division, cellularization, and cell fate determination.

Helicases form a large group of motor proteins that affect many processes of nucleic acid metabolism by catalyzing the separation of complementary strands of double-stranded (ds) RNA/DNA or remodeling of protein–RNA/DNA complexes (Fairman-Williams *et al.*, 2010). Eukaryotic helicases belong to two superfamilies that share at least eight sequence motifs (i.e. I, Ia, Ib, II, III, IV, V, and VI). According to their sequences, as well as structural and mechanistic features, helicases can be classified into 12 families or groups. Of them, DEAD-box, DEAH/RHA, NS3/NPH-II, Ski2-like, and RIG-I-like families exhibit a strong preference for unwinding RNA duplexes. The DEAH/RHA RNA helicases contain the helicase core domains (i.e. DEXDc and HELICc) at their N-terminus, and the helicase-associated domain (HA2) and oligonucleotide/oligosaccharide-binding (OB)-fold domains at the C-terminus. Crystal structure analyses have revealed that the C-terminus of DEAH/RHA RNA helicases consists of the winged helix-fold, ratchet, and OB-fold domains (Walbott *et al.*, 2010). These domains function in RNA splicing, ribosome biogenesis, and transcription regulation by promoting the rearrangement of structured RNAs and remodeling of RNA–protein complexes (Fairman-Williams *et al.*, 2010; Jarmoskaite and Russell, 2014).

In yeast, several DEAH/RHA RNA helicases regulate pre-mRNA splicing, such as PRP2, PRP16, PRP22, and PRP43, which function at different stages of splicing (Will and Lührmann, 2011; Hoskins and Moore, 2012). PRP2 and PRP16 are involved in the release of splicing proteins during splicing. PRP22 is required for the second transesterification step and release of mature mRNA. PRP43 affects the release of the intron lariat by helping to disassemble the spliceosome after splicing reactions have been completed (Jarmoskaite and Russell, 2014). The Arabidopsis genome encodes 20 putative DEAH/RHA RNA helicases, but the plant developmental functions for these enzymes have not been determined, except for ENHANCED SILENCING PHENOTYPE 3/RADIAL SWELLING 12 (EPS3/RSW12; At1g32490), FASCIATED STEM 4 (AtFAS4; At1g33390), ABA OVERLY SENSITIVE 6 (ABO6; At5g04895), CLUMSY VEIN (CUV; At5g13010), and ROOT INITIATION DEFECTIVE 1 (RID1; At1g26370). EPS3 is a putative homolog of yeast PRP2, which is required for RNA processing and plays an important role in the regulation of embryo development, flowering, and temperature-sensitive seedling growth (Tzafrir *et al.*, 2004; Herr *et al.*, 2006; Howles *et al.*, 2016). Overexpression of *AtFAS4* in Arabidopsis results in the development of a fasciated stem (Pogorelko *et al.*, 2008). ABO6 contributes to abscisic acid-regulated production of reactive oxygen species in Arabidopsis roots by splicing the pre-mRNA of mitochondrial electron transport chain complex I genes (He *et al.*, 2012). CUV, which is an Arabidopsis DEAH-box PRP16 homolog, functions in auxin-mediated development (Tsugeki *et al.*, 2015). Although the RID1 amino acid sequence is highly similar to the yeast PRP22 sequence, it cannot complement the cold-sensitive phenotype of the yeast *prp22* mutant (Ohtani *et al.*, 2013). During pre-mRNA splicing in Arabidopsis, RID1 is involved in the recognition of the splicing site and removal of the intron, which influences meristem maintenance, leaf morphogenesis, and root morphogenesis under high temperature conditions (Ohtani *et al.*, 2013). Additionally, mutations in *RID1* lead to abnormal cellular specification in mature FGs, including the development of similar-sized synergid and egg cell nuclei, unfused polar nuclei, enlarged and protruded antipodal cells, and fused antipodal nuclei. These observations in *rid1* mutants suggest the importance of RID1 during FG development.

RNA biogenesis is believed to be crucial for FG development (Shi and Yang, 2011). For example, SLOW WALKER 1 (SW1), SLOW WALKER 3/Arabidopsis RNA Helicase 36 (SW3/AtRH36; a DEAD box helicase), YAOZHE (YAO), and NUCLEOLAR FACTOR 1 (NOF1) function in mitotic progression during FG development by regulating 18S pre-rRNA processing and rRNA expression (Shi *et al.*, 2005; Harscoët *et al.*, 2010; Huang *et al.*, 2010; Li *et al.*, 2010; Liu *et al.*, 2010). In addition, *Gametophyte Defective 1* (*GAF1*), which encodes a putative subunit of RNase P, is responsible for the processing of tRNA and transcription of small non-coding RNAs. The *gaf1* mutant displays retarded FG development (Wang *et al.*, 2012).

Messenger RNA splicing is a critical step during gene expression in eukaryotes. ATROPUS, GAMETOPHYTIC FACTOR 1 (GFA1)/CLOTHO/VAJRA-1/MEE5, and LACHESIS are yeast homologs of the components of the mRNA splicing machinery in Arabidopsis (Feldmann *et al.*, 1997; Pagnussat *et al.*, 2005; Coury *et al.*, 2007; Groß-Hardt *et al.*, 2007; Moll *et al.*, 2008; Liu *et al.*, 2009; Yagi *et al.*, 2009). Loss-of-function mutations to these proteins result in altered size and shape of floral organs, defects in FG development, changes to gametic cell fate, and arrested embryo and endosperm development. For example, GFA1 is the Arabidopsis homolog of U5-116kD and SNU114, which are U5 small nuclear ribonucleoprotein (snRNP) particle components required for pre-mRNA splicing in humans and yeast, respectively (Brenner and Guthrie, 2006; Frazer *et al.*, 2008). In the T-DNA insertion mutant *gfa1*, the FGs contain an abnormal number of nuclei and cellular structures during the early developmental stages, or the mature ovules lack FGs (Coury *et al.*, 2007). *sgt13018* is a partial loss-of-function *gfa1* mutant that exhibits delayed FG development after the FG5 stage. In addition, the fusion of polar nuclei during the late FG developmental stages is impaired in this mutant (Liu *et al.*, 2009). In *clotho* mutants, which carry a single base-pair mutation in *GFA1*, the synergids and the central cell adopt an egg cell fate (Moll *et al.*, 2008). GFA1 interacts with AtBRR2 and AtPRP8, which are two putative Arabidopsis homologs of U5 snRNP components (Liu *et al.*, 2009). The results of these studies suggested that U5 snRNP-involved pre-mRNA splicing might be important for FG development. However, the mechanism underlying this process remains to be investigated.

In this study, we determined that the DEAH-box RNA helicase RID1 is required for FG development. Furthermore, we demonstrated that RID1 interacts with GFA1, and this interaction is involved in FG development through the pre-mRNA splicing of relevant genes. Our results provide new information regarding the regulation of FG development in Arabidopsis.

## Materials and methods

### Plant materials and growth conditions

T-DNA insertion lines of *Arabidopsis thaliana* (GABI_310A05, GABI_730B12, and SALK_025707) were ordered from The Nottingham Arabidopsis Stock Centre (NASC) and the Arabidopsis Biological Resource Center (ABRC). The genotypes of T-DNA insertion line plants and their progenies were determined by a PCR-based method using specific primers: RID1LP1and RID1RP1 for GABI_730B12, RID1LP2 and RID1RP2 for GABI_310A05, and GABI T-DNA specific primer T-DNALB. All primers used in this study are listed in Supplementary Table S1 at *JXB* online.

Arabidopsis seeds were surface-sterilized with 2.6% (v/v) sodium hypochlorite for 8–10min, and then washed five or six times in sterilized water and plated on Murashige and Skoog agar plates. For antibiotic selection of transgenic seeds, 50mg l^–1^ kanamycin or 20mg l^–1^ hygromycin was added as required. After cold treatment for 3 d at 4 °C in the dark, they were transferred to a growth room at 22±2 °C in a 16/8h light/dark cycle. Arabidopsis transformation was performed by *Agrobacterium tumefaciens*-mediated infiltration. Tobacco (*Nicotiana benthamiana*) plants were grown in a 12/12h light/dark cycle at 25 °C in a greenhouse.

### Observation of FG development

The development of ovules was observed according to the method described by Shi *et al.* (2005). The pistils were fixed in 4% glutaraldehyde overnight at room temperature. After conventional ethanol series dehydration, the fixed materials were cleared in 2:1 (v/v) benzyl benzoate:benzyl alcohol for 5h. The ovules dissected from the pistils were observed with a Zeiss LSM510 META confocal laser scanning microscope (Zeiss, Jena, Germany) with a 488-nm excitation argon laser and an LP 530 emission filter.

### RID1 helicase activity assays

The cDNA sequence of RID1 was cloned into the bacterial expression vector pGEX-4T-1 at the EcoRI and XhoI sites to create pGEX-4T-RID1. The pGEX-4T-RID1 plasmid was transformed into *Escherichia coli* BL21 (DE3) cells, and the recombinant GST-RID1 protein was purified using glutathione-Sepharose beads (GE Healthcare, Chalfont St. Giles, Buckinghamshire, UK) column chromatography following the manufacturer’s instructions. After confirmation by sodium dodecyl sulfate polyacrylamide gel electrophoresis (SDS-PAGE), purified GST-RID1 was used for all helicase activity assays.

A molecular beacon helicase assay was performed according to the description by Belon and Frick (2008) and Mukherjee *et al.* (2012). RNA oligonucleotides were ordered from Takara Biotechnology Co., Ltd. (Dalian, China), and the fluorescent strand was modified with Cyanine 5 (Cy5) at the 3′ end and Black Hole Quencher (BHQ) at the 5′ end. The dsRNA substrates were prepared by combining unlabeled and labeled oligonucleotides at a 2:1 molar ratio in 40mM Tris-HCl (pH 7.5) and 0.5mM MgCl_2_, placing the reaction in 95 °C water, and allowing it to cool to room temperature. The unwinding reaction system contained 2mM MgCl_2_, 2mM DTT, 0.1mM BSA, 1 μM enzyme, 2mM ATP, 8nM dsRNA substrate, 4U RNAase inhibitor, and 50mM Tris-HCl (pH 7.5) at 22 °C. Fluorescence was detected for excitation/emission at 643/667nm using a Synergy MX Multi-Mode Microplate Reader (BioTek Instruments Inc., Winooski, VT, USA) and was recorded in each well every 30s. This experiment was repeated three times, and the fluorescence intensity was recorded as arbitrary units (a.u.).

### Yeast two-hybrid assay

The full-length *RID1* cDNA and fragments of *RID1* were cloned into the pGADT7 vector (Clontech, Palo Alto, CA, USA) at the EcoRI and XhoI sites to create the pAD-RID1 bait vector. RID1 fragments with mutant amino acid residues were created using a TaKaRa MutanBEST Kit (TaKaRa, Dalian, China). The pBD-GFA1 prey vector was a generous gift from Dr Yang Wei-Cai (Chinese Academy of Sciences, Beijing, China). Yeast transformation was carried out using a kit (Clontech, Palo Alto, CA, USA) according to the manufacturer’s protocol. The yeast strain used was *Saccharomyces cerevisiae* Y2H gold (Clontech, Palo Alto, CA, USA). Transformed cells were plated on SD-Trp-Leu/X-α-Gal/AbA for screening of positive colonies, and incubated at 30 °C for 3–5 d. Host yeast cells were transformed with the Empty AD/BD vectors used as negative controls.

### Protein pull-down assay

The amplified full-length *RID1* cDNA was digested by EcoRI and XhoI and inserted into the pET-28a-c(+) vector at te EcoRI and SalI sites to create pET-28a-RID1. The full-length *GFA1* cDNA was amplified by the primers GFA1-gst-up and GFA1-gst-down and then cloned into pGEX-4T-1 at the *BamH*I and *Xho*I sites to yield pGEX-4T-GFA1. The constructs pET-28a-RID1 and pGEX-4T-GFA1 were transformed into *E. coli* BL21 (DE3) cells. His-RID1 fusion protein was purified by batch elution from affinity Ni Sepharose™ 6 Fast Flow beads (GE Healthcare, Chalfont St. Giles, Buckinghamshire, UK). GST and GST-GFA1 fusion proteins were purified by batch elution from affinity glutathione-Sepharose 4B beads (GE Healthcare, Chalfont St. Giles, Buckinghamshire, UK). Protein glutathione-Sepharose 4B beads were equilibrated with buffer (50mM Tris-HCl, pH 7.5; 100mM NaCl; 1mM EDTA Free Protease Inhibitor Cocktail) (Roche, Indianapolis, IN, USA) and incubated with GST or GST-GFA1 fusion protein at 4 °C for 30min with end-to-end shaking. After incubation, beads were washed three times with the rinse buffer (50mM Tris-HCl, pH 7.5; 100mM NaCl; 1mM EDTA Free Protease Inhibitor Cocktail). His-RID1 fusion protein was then incubated with glutathione-Sepharose 4B beads at 4 °C for 1–2h with end-to-end shaking. After centrifugation, the beads were washed three times with wash buffer (50mM Tris-HCl, pH 7.5; 150mM NaCl; 25mm imidazole; 1mM EDTA Free Protease Inhibitor Cocktail) and then mixed with 50 μl of 2× SDS sample buffer. Twenty microliters of the supernatant were then loaded onto an SDS-PAGE gel, and immunoblotting was performed using monoclonal anti-HIS antibody (1:1000; Sigma).

### Bimolecular fluorescence complementation

The full-length coding sequences of RID1 and GFA1 were fused with N and C parts of yellow fluorescent protein (YFP), respectively. Primers for generating these constructs are listed in Supplementary Table S1. Agrobacteria (GV3101) harboring the constructs 35S::RID1-YFP_N_ and 35S::GFA1-YFP_C_ were used to infiltrate the abaxial side of 1-month-old *N. benthamiana* leaves. After 3 d, fluorescence was detected via confocal microscopy.

### Analysis of pre-mRNA splicing and gene expression

The *rid1-2*/+ and *gfa1-1/+* pistils of 9- or 10-stage flowers were collected for total RNA extraction using the RNeasy Plant Mini Kit (Qiagen, Hilden, Germany) according to the manufacturer’s instructions. After genomic DNA contamination was removed, total RNA (2 µg) was used for reverse transcription using a kit (Promega, Madison, WI, USA). Quantitative real-time PCR (qRT-PCR) analysis was performed as previously described (Li *et al.*, 2011). Quantitative variation in the different replicates was calculated using the delta-delta threshold cycle relative quantification method. Amplification of TUBULIN2 was used as an internal control. The results are displayed as the mean ±SEM of three individual experiments. All primers are listed in Supplementary Table S1.

The PCR conditions for At1g09620 and At3g55630 were: 94 °C for 2min, followed by 38 cycles of 94 °C for 30s, 58 °C for 30s, and 70 °C for 30s, and completed at 72 °C for 10min. For At2g01730, PCR was performed at 94 °C for 2min, followed by 35 cycles of 94 °C for 30s, 58 °C for 30s, and 70 °C for 30s, and completed at 72 °C for 10min. For At4g39260, the reaction was performed at 94 °C for 2min, followed by 32 cycles of 94 °C for 30s, 60 °C for 30s, and 70 °C for 30s, and completed at 72 °C for 10min. The amplified PCR products were resolved by electrophoresis on 1.5% agarose gels. All primers are listed in Supplementary Table S1.

## Results

### Defective RID1 retards FG development

**Fig. 1.**
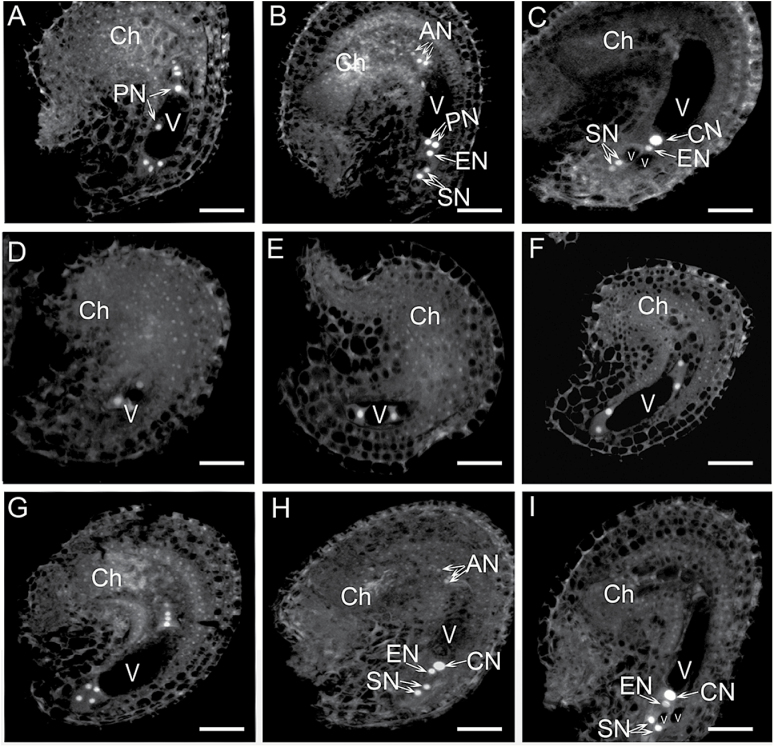
FG development in stage 12 flowers of wild-type and *rid1-2/+* mutant plants. (A–C) Three stages of FG development. The embryo sacs at the FG 5 (A), FG6 (B), and FG7 (C) stages in pistils of wild-type stage 12 flowers. (D–I) Six stages of FG development. The embryo sacs at the FG2 (D), FG3 (E), FG4 (F), FG5 (G), FG6 (H), and FG7 (I) stages in pistils of *rid1-2/+* stage 12 flowers. The developmental stages of FG were determined according to Christensen *et al.* (1997), and the nuclear position was examined by detecting their autofluorescence (for details see the Methods section). AN, antipodal nucleus; Ch, chalazal end; CN, central cell nucleus; EN, egg cell nucleus; PN, polar nucleus; SN, synergid nucleus; V, vacuole. Scale bars = 10 μm.

A previous study revealed that a mutation in *RID1* caused abnormal cell specification of synergids, egg cells, and antipodal cells in mature FGs (Ohtani *et al.*, 2013). In this study, we used two independent *RID1* knockout lines mutated by T-DNA insertion [i.e., GABI_730B12 (*rid1-2*/*+*) and GABI_310A05 (*rid1-3*/*+*)] to confirm that RID1 is required for FG development (Supplementary Fig. S1). We examined the T-DNA insertion line *rid1-2*/*+* to further analyze the defects in FG development in *rid1* mutants. Stage 12 flowers in *rid1-2*/+ mutant and wild-type plants were examined according to the methods described by Shi *et al.* (2005). The development of FGs in wild-type Arabidopsis plants can be divided into eight distinct stages based on FG morphological changes (Christensen *et al.*, 1997). After the megaspore mother cell undergoes meiosis, the megaspore at the chalazal end gives rise to a mature FG following three rounds of mitosis. One-, two-, four-, and eight-nucleate FGs were represented by the FG1, FG2/FG3, FG4, and FG5 stages, respectively (Supplementary Fig. S2). In the majority of pistils from wild-type stage 12 flowers, we observed stages FG5–7 (Fig. 1A–C). In the pistils of *rid1-2*/+ stage 12 flowers, approximately half of the FGs were at the FG5–7 stages. The remaining stages ranged from FG2 to FG4 (Fig. 1D–I), indicating that mitotic activities during FG development were retarded in the *rid1* pistils.

The development of FGs within the same wild-type pistil was more-or-less synchronous during a single developmental stage or in two consecutive stages (Table 1) (Christensen *et al.*, 1997; Shi *et al.*, 2005). Thus, we investigated the developmental synchrony of *rid1-2*/+ FGs in pistils at different stages, as described by Shi *et al.* (2005). We observed that the synchrony of FG development in the *rid1-2*/+ mutant was disturbed. More than four FG stages were detected in each *rid1-2*/+ pistil (from P3 to P7) (Table 2), compared with two FG stages in each wild-type pistil (from P4 to P6) (Table 1). Only seven of 44 FGs were at the FG7 stage in the sixth pistil of *rid1-2*/+ inflorescences, whereas 14 of 27 were at the FG7 stage in the corresponding pistil of the wild-type inflorescences (Tables 1 and 2). These results indicated that a *RID1* mutation not only retarded the progression of nuclear division, it also disturbed the synchrony of FG development.

**Table 1.** Synchrony of FG development in wild-type pistils

**Pistil position** ^**§**^	FG developmental stages*							**Total FGs**
	**FG1**	**FG2**	**FG3**	**FG4**	**FG5**	**FG6**	**FG7**	
P1	22							22
P2	8	12	8	4				32
P3			11	4	7	3		25
P4					5	6		11
P5						23	5	28
P6						13	14	27

^§^ P1 to P5 indicate the position of pistils at inflorescence. P6 is the pistil of the stage 12 flower at the base of the inflorescence.

* FG stages are defined according to Christensen *et al.* (1997).

**Table 2.** Synchrony of FG development in rid1-2/+ pistils

**Pistil position** ^**§**^	FG developmental stages*							**Total FGs**
	**FG1**	**FG2**	**FG3**	**FG4**	**FG5**	**FG6**	**FG7**	
P1	18							18
P2	17	4						21
P3	15	15	5	5				40
P4	10	4	6	10				30
P5		2	3	15	6	2	2	30
P6		1	3	8	10	15	7	44
P7		2	1	2	1	3	8	17

^§^ P1 to P7 indicate the position of pistils at inflorescence. P7 is the pistil of the stage 12-flower at the base of the inflorescence.

* FG stages are defined according to Christensen *et al.* (1997).

Some developmentally delayed FGs in the pistils of mutant plants can still be functional (Shi *et al.*, 2005; Li *et al.*, 2009). To determine whether the *rid1* FG is functional, delayed pollination tests were completed according to the method described by Shi *et al.* (2005). The stage 12 *rid1-2*/+ flowers were emasculated, and then pollinated with wild-type pollen grains 72h later. The seedling genotypes of the progenies were determined using a PCR-based method. In total, nine heterozygous plants (*rid1-2*/+) were detected among 171 seedlings, indicating that some ovules could develop into functional FGs for fertilization under delayed pollination conditions (Supplementary Table 2).

### RID1 exhibits RNA helicase activity

**Fig. 2.**
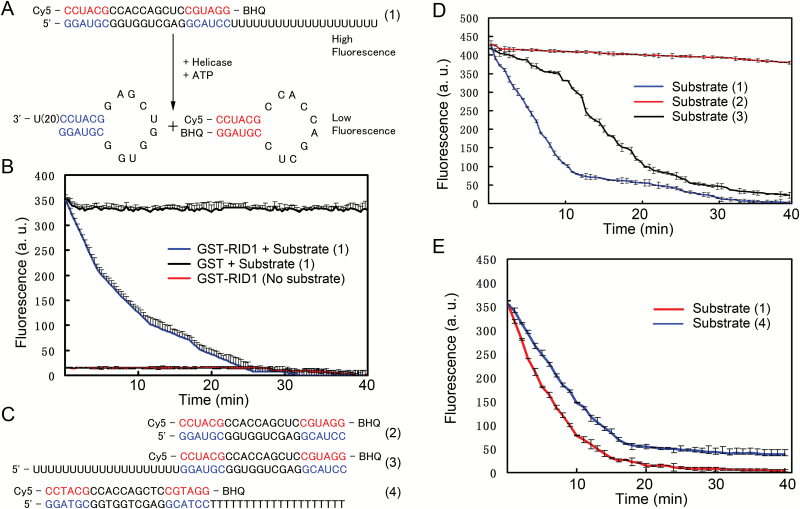
Helicase activity of RID1 unwinding dsRNA/DNA according to the molecular beacon helicase assay (MBHA). (A) The fluorescent strand in the 3′ overhanging RNA substrate (1) containing a 20-bp hanging tail was modified with Cy5 at the 3′ end and BHQ at the 5′ end. The fluorescence of Cy5 was quenched after the substrate RNA double strands were unwound and the fluorescent strand formed a stem-loop structure, which placed Cy5 near BHQ. (B) The RID1-GST fusion protein, but not GST alone, decreased the intensity of fluorescence in the MBHA system in which substrate (1) was used. A reaction system lacking RNA substrate was used as the control. a.u., arbitrary units.(C) The fluorescent strands of the blunt-ended RNA substrate (2), 5′ overhanging RNA substrate (3), and 3′ overhanging DNA substrate (4) were modified with Cy5 at the 3′ end and BHQ at the 5′ end. (D) RID1 could unwind RNA double strands from the 3′ to 5′ and 5′ to 3′ directions. The components of the MBHA system are described in the Methods section. Error bars represent the standard deviation of three independent experiments. a.u., arbitrary units. (E) RID1 could unwind RNA and DNA double strands. The components of the MBHA system are described in the Methods section. Error bars represent the standard deviation of three independent experiments. a.u., arbitrary units.

The results of a phylogenetic analysis indicated that RID1 is highly homologous to several proteins from different species (Supplementary Fig. S3). An alignment of the protein sequences revealed that RID1 contains 12 conserved motifs (I, Ia, Ib, Ic, II, III, IV, IVa, V, Va, Vb, and VI) in the N-terminus (Supplementary Fig. S4). However, *RID1* could not complement the cold-sensitive phenotype of yeast *prp22* mutants (Ohtani *et al.*, 2013). To verify the role of RID1 in unwinding dsRNA/DNA, we purified a glutathione S-transferase (GST)-RID1 fusion protein from *E. coli*, and analyzed its helicase activity using the *in vitro* molecular beacon helicase assay (Belon and Frick, 2008; Mukherjee *et al.*, 2012). Three types of dsRNA substrates were used to determine the direction of RID1 translocation (i.e. 3′- and 5′-overhanging dsRNA and blunt-ended dsRNA) (Fig. 2A, C). We determined that RID1 could unwind dsRNA in both directions, with a preference for 3′ to 5′ unwinding. Minimal unwinding activity was observed with blunt-ended dsRNA (Fig. 2B–D). In addition, RID1 could also unwind dsDNA, but at lower activity levels than with dsRNA (Fig. 2E).

### RID1 interacts with GFA1 in the nucleus

**Fig. 3.**
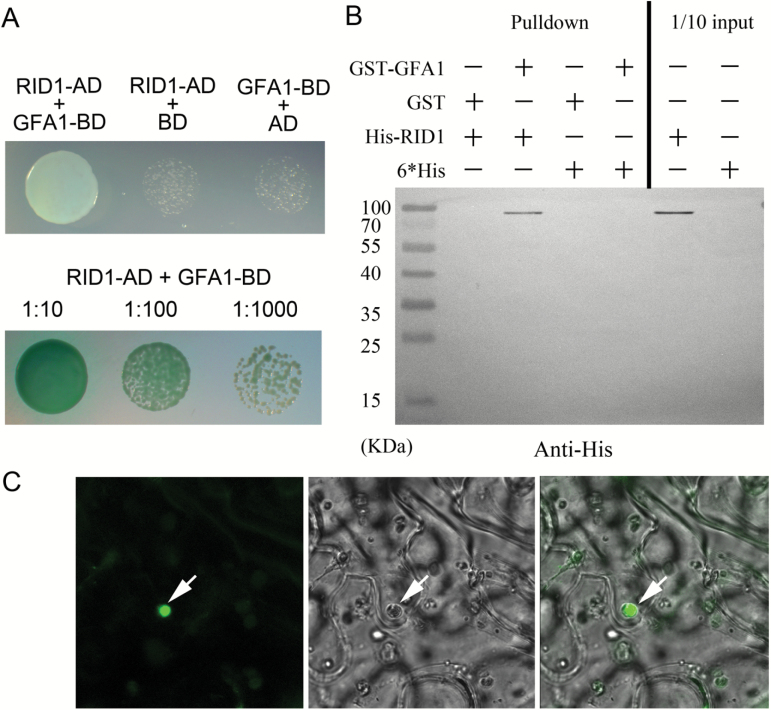
RID1 interacting with GFA1 in the nucleus. (A) RID1 interacting with GFA1 in yeast cells. The transformed yeast cells were used to inoculate synthetic drop-out selection medium that lacked Trp, Leu, His, and Ade. (B) Pull-down analysis revealing RID1 is directly associated with GFA1. (C) Fluorescence was detected in the nucleus of tobacco leaf epidermal cells co-transformed with 35S::*RID1-YFP*_*N*_ and 35S::*GFA1-YFP*_*C*_.

RID1 is a DEAH-box RNA helicase and homolog of yeast PRP22, which affects yeast cell cycle progression and pre-mRNA splicing by interacting with the snRNP spliceosome (Hwang and Murray, 1997; Wang and Brendel, 2004). SNU114/U5-116kD, which forms a complex with PRP8 and BRR2, is an integral protein of the U5 snRNP, and is important for regulating spliceosomal dynamics (Frazer *et al.*, 2008; Hoskins and Moore, 2012). Arabidopsis GFA1, AtBRR2, and AtPRP8 are the homologs of yeast SNU114, BRR2, and PRP8, respectively. GFA1 is believed to be involved in pre-mRNA splicing through interactions with AtBRR2a and AtPRP8a (Liu *et al.*, 2009). Several *GFA1* mutants (*sgt13018*, *gfa1-1*, *gfa1-2*, *gfa1-3*, and *clotho*) from different sources exhibited retarded FG development and abnormal cellular specification in mature FGs (Feldmann *et al.*, 1997; Pagnussat *et al.*, 2005; Coury *et al.*, 2007; Groß-Hardt *et al.*, 2007; Moll *et al.*, 2008; Liu *et al.*, 2009), which is consistent with the FG phenotypes of *rid1* mutants. The *RID1* and *GFA1* expression patterns were also similar during Arabidopsis development (Supplementary Fig. S5) (Schmid *et al.*, 2005). Additionally, RID1 and GFA1 are both localized in the nucleus (Moll *et al.*, 2008; Ohtani *et al.*, 2013). Thus, we hypothesized that RID1 and GFA1 may interact to regulate FG development. To test this hypothesis, we completed a yeast two-hybrid analysis to characterize the interaction between RID1 and GFA1. The full-length *RID1* and *GFA1* coding sequences were cloned into pGADT7 and pGBKT7 as ‘prey’ and ‘bait’, respectively. On minimal medium lacking tryptophan, leucine, histidine, and adenine (SD/−Trp/−Leu/−His/−Ade), the yeast cells co-transformed with the bait pBD-GFA1 and prey pAD-RID1 constructs grew well and could degrade the substrate X-α-gal to produce blue colonies (Fig. 3A). These results suggested that GFA1 physically interacts with RID1 in yeast cells.

To confirm the physical interaction between RID1 and GFA1, we expressed and purified hexa-histidine-tagged RID1 (His-RID1) and GST-tagged GFA1 (GST-GFA1) for *in vitro* pull-down assays. Purified GST-GFA1 was added to the affinity column, and after washing, purified His-RID1 was added to the same column (Fig. 3B). After washing, the proteins bound to the column were eluted and examined by immunoblotting with antibodies specific for the His-tag. We observed that His-RID1 was pulled down by GST-GFA1 after co-incubation. GST alone did not interact with His-RID1.

To further confirm an interaction between RID1 and GFA1 *in vivo*, we completed bimolecular fluorescence complementation assays. RID1 and GFA1 were fused to the N- and C-terminal parts of yellow fluorescent protein (YFP), respectively, to generate 35S::*RID1-YFP*_*N*_ and 35S::*GFA1-YFP*_*C*_. Tobacco (*N. benthamiana*) leaves were co-transformed with the constructs, and strong fluorescence signals were detected in the nucleus of epidermal cells 3 d later (Fig. 3C). No fluorescence was observed in tobacco leaves co-transformed with 35S::*RID1-YFP*_*N*_ and 35S::*YFP*_C_, or 35S::*GFA1-YFP*_*C*_ and 35S::*YFP*_*N*_ (data not shown). These results indicated that RID1 interacts with GFA1 in the nucleus.

### Residues Y266 and T267 in RID1 are important for the interaction with GFA1

**Fig. 4.**
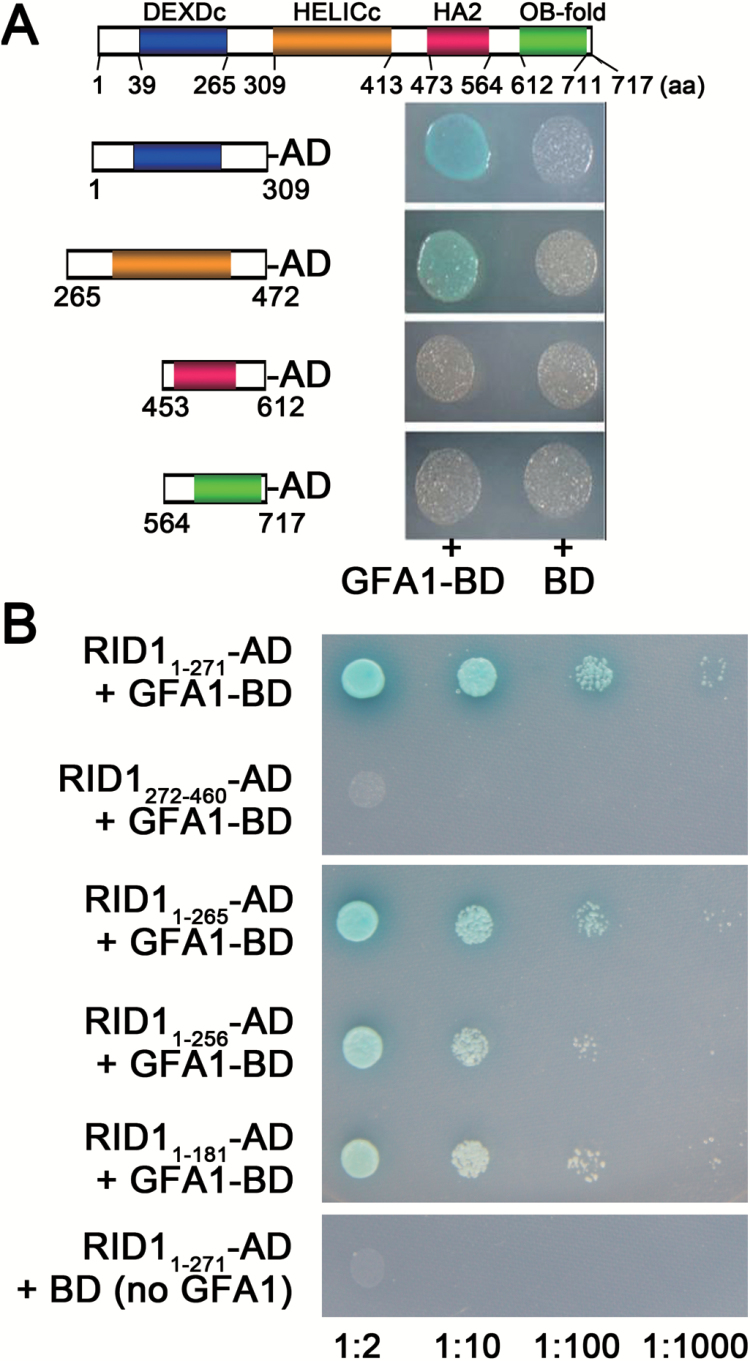
Determining the RID1 fragments that interacted with GFA1 using yeast two-hybrid analyses. (A) Interactions between GFA1-BD and a series of AD-prey carrying N- or C-terminal RID1 domains. (B) Interactions between GFA1-BD and a series of AD-prey carrying RID1 with C-terminal deletions.

Analysis of the Conserved Domain Database (http://www.ncbi.nlm.nih.gov/cdd) (Marchler-Bauer *et al.*, 2015) revealed that RID1 has four domains, namely DEXDc, HELICc, HA2, and OB-fold (Fig. 4A). To determine which RID1 region interacts with GFA1, we used yeast two-hybrid analyses to assess the interaction between GFA1 and the RID1 domains. We divided RID1 into four segments, with each containing one domain. We then investigated the interaction between these four segments and GFA1 using a yeast two-hybrid system, and determined that the GFA1-binding RID fragments were confined to amino acids 1–309 and 265–472 (Fig. 4A). Furthermore, we observed that RID1 amino acids 1–271 (RID1_1–271_) interacted with GFA1, while RID1_272–460_ did not (Fig. 4B). In addition, the RID1_1–181_ and RID1_1–256_ fragments exhibited negligible interactions with GFA1 (Fig. 4B). These results suggested that the RID1_257–271_ sequence is important for the interaction between GFA1 and RID1.

**Fig. 5.**
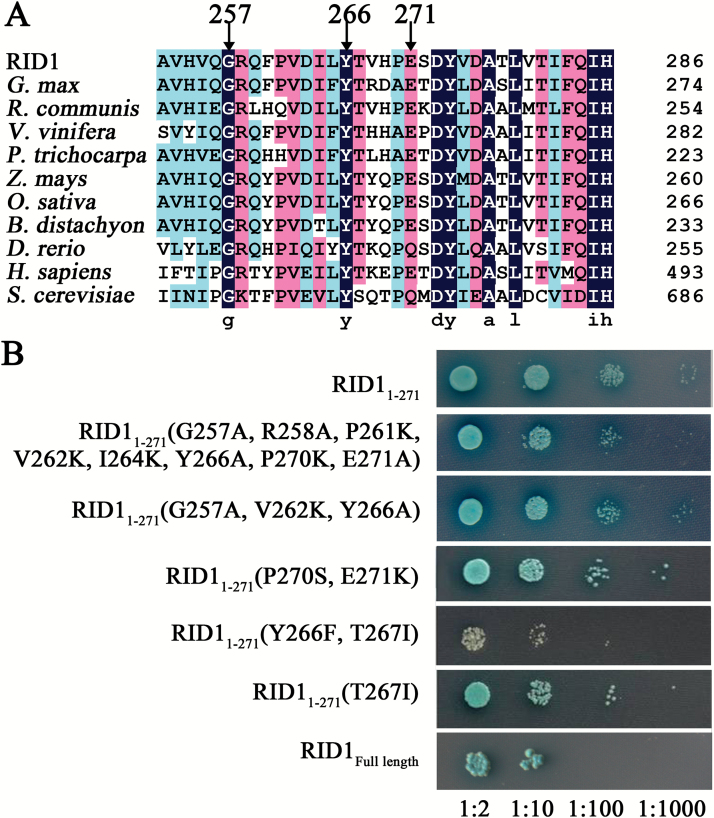
Mapping the key RID1 sites that interacted with GFA1 using yeast two-hybrid analyses. (A) Sequence alignment involving RID1 and its homologs in *Glycine max* (XP_003520120.1), *Ricinus communis* (XP_002512067.1), *Vitis vinifera* (XP_002282341.2), *Populus trichocarpa* (XP_002311345.1), *Zea mays* (NP_001147775.1), *Oryza sativa* (BAD35264.1), *Brachypodium distachyon* (XP_003577484.1), *Danio rerio* (NP_001093527.3), *Homo sapiens* (EAW51679.1; DHX8), and *Saccharomyces cerevisiae* (CAA41530.1; PRP22). (B) Effects of substitutions of conserved amino acids on the interaction between RID1_1-271_-AD and GFA1-BD in a yeast two-hybrid system.

Sequence alignments involving RID1 and its homologs from plant, yeast, and animal species revealed that the residues G257, R258, P261, V262, I264, Y266, T267, and E271 were conserved (Fig. 5A). To determine the role of these conserved amino acids in the interaction between RID1 and GFA1, we replaced them with other residues and analyzed the interaction between the mutant RID1_1-271_-AD and wild-type GFA1-BD using yeast two-hybrid analysis (Fig. 5B). We observed that the interaction between RID1_1-271_ and GFA1 was weakened when all conserved residues were substituted (i.e. G257A, R258A, P261K, V262K, I264K, Y266A, P270K, and E271A). This result was consistent with those for the mutant RID1_1-271_ (i.e. G257A, V262K, and Y266A or P270S and E271K). The interaction between RID1_1-271_-AD and GFA1-BD was obviously weakened when Y266 and T267 were mutated (Fig. 5B). Furthermore, we determined that the interaction between RID1_1-271_-AD and GFA1-BD was stronger after the T267I mutation or when Y266 (but not T267) was mutated [RID1_1-271_ (G257A, V262K, and Y266A)] than after the double mutation of Y266F and T267I (Fig. 5B). These results suggested that Y266 and T267 are critical for the interaction between RID1 and GFA1.

**Fig. 6.**
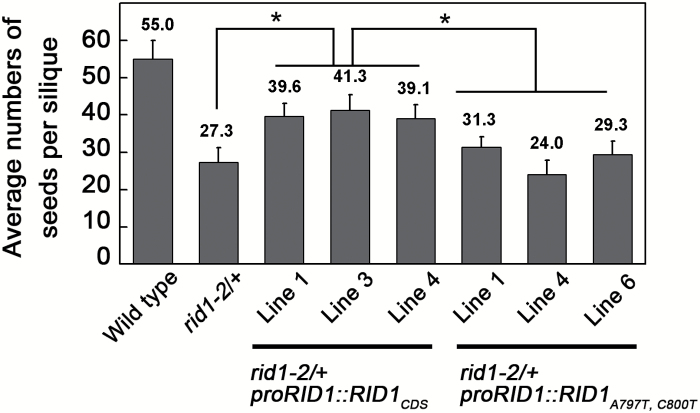
Analysis of the average number of seeds per siliques. Seeds in five sequential siliques from six independent wild-type, *rid1-2*/+ mutant, *rid1-2*/+ *proRID1::RID1*_*CDS*_, and *rid1-2*/+ *proRID1::RID1*_*A797T,C800T*_ plants were counted. Data are presented as the mean ± standard deviation. Numbers above columns correspond to the average number of seeds per silique. * significant difference (Student’s *t*-test, *P* < 0.01).

To clarify the effects of RID1 residues Y266 and T267 on the regulation of FG development in Arabidopsis, we attempted to complement the *rid1-2*/+ mutant using a RID1 with two amino acid mutations (Y266F and T267I). A construct consisting of the promoter sequence (1504bp upstream of the ATG start codon) fused to the *RID1* coding sequence with two base mutations (A797T and C800T), designated as *proRID1*::*RID1*_*A797T,C800T*_, was introduced into the *rid1-2*/+ mutant by *Agrobacterium*-mediated transformation. Additionally, a control construct comprising the promoter sequence fused to the *RID1* coding sequence, designated as *proRID1*::*RID1*_*CDS*_, was transformed into the *rid1-2*/+ mutant. Three randomly selected transgenic lines of these two transformations were used for further analysis. We counted the number of seeds in siliques from wild-type, *rid1-2*/+ mutant, *rid1-2*/+ *proRID1::RID1*_*CDS*_, and *rid1-2*/+ *proRID1::RID1*_*A797T,C800T*_ plants. Our results revealed that the *rid1-2*/+ *proRID1::RID1*_*CDS*_ transgenic plants produced many more seeds than the *rid1-2*/+ mutant or *rid1-2*/+ *proRID1::RID1*_*A797T,C800T*_ plants (Fig. 6). In addition, there were no important differences in seed numbers between the *rid1-2*/+ mutant and *rid1-2*/+ *proRID1::RID1*_*A797T,C800T*_ plants. These results indicated that the RID1 Y266 and T267 residues are involved in the interaction between RID1 and GFA1, and that this interaction is critical for FG development.

### RID1 and GFA1 are involved in pre-mRNA splicing of the genes required for FG development

**Fig. 7.**
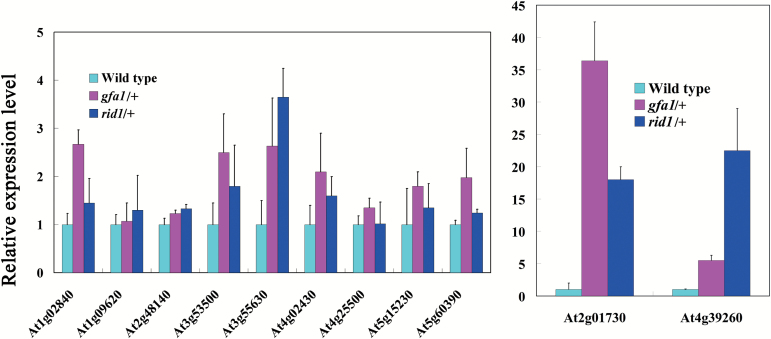
Some genes show abnormal pre-mRNA splicing in both *gfa1-1*/+ and *rid1-2*/+ mutants. The abnormal retention of first introns in pre-mRNA of RNA splicing-related and female gametophyte development-related genes in *rid1-2*/+ and *gfa1-1*/+ mutants were detected by qRT-PCR analysis. The expression of *TUBULIN2* was used as the internal control. All experiments were repeated three times.

**Fig. 8.**
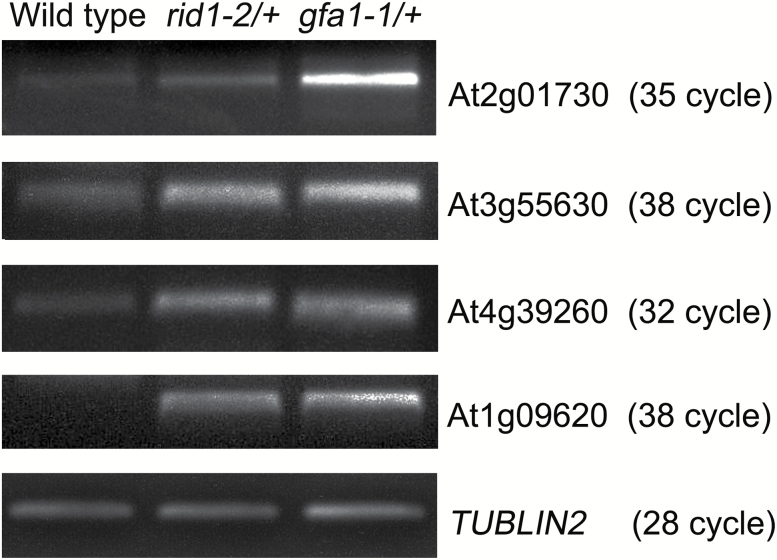
Semi-quantitative RT-PCR analysis of the retention of the first intron in pre-mRNAs in *rid1-2*/+ and *gfa1-1*/+ mutant pistils. Numbers in brackets refer to the cycle numbers for the corresponding PCR analysis.

Nine *rid1* mutant genes have been identified with modified pre-mRNA alternative splicing patterns during callus initiation of hypocotyl explants under high-temperature conditions (Ohtani *et al.*, 2013). Because several genes required for FG development have been identified in Arabidopsis (Pagnussat *et al.*, 2005; Yang *et al.*, 2010; Drews and Koltunow, 2011), we hypothesized that RID1 and GFA1 regulate FG development through pre-mRNA splicing of these genes. Therefore, we used qRT-PCR to analyze the retention of the first intron in the transcripts of these genes in the pistils of *rid1-2/+* and *gfa1-1*/+ mutants. Because it is difficult to obtain homozygous *rid1* and *gfa1* mutants, we sampled the pistils of heterozygous mutants for pre-mRNA splicing analysis. Total RNA was extracted from equal numbers of wild-type and mutant ovules, with the wild-types used as controls. The qRT-PCR analysis revealed abnormal retention of the first introns in the mature transcripts of alternative splicing-related genes and FG development-related genes in the *rid1-2/+* and *gfa1-1/+* (SALK_025707) mutants (Coury *et al.*, 2007) (Table 3, Fig. 7). This suggested that RID1 and GFA1 are involved in splicing common pre-mRNAs during the regulation of FG development. *TRANSLATIONAL ELONGATION FACTOR 1α* (*AtEF1α*; At5g60390) is a housekeeping gene in Arabidopsis (Axelos *et al.*, 1989), and *GAST1 PROTEIN HOMOLOG 4* (*GASA4*; AT5G15230) is a gibberellin-induced gene involved in gibberellin responses and signaling in reproductive development (Roxrud *et al.*, 2007; Rubinovich and Weiss, 2010). Abnormal retention of the first introns in the *AtEF1α* and *GASA4* pre-mRNAs was detected in the *rid1-2*/+ and *gfa1-1*/+ mutants (Table 3, Fig. 7). Therefore, RID1 and GFA1 were involved in the splicing of genes affecting basic biochemical processes in addition to FG development and alternative splicing. Furthermore, semi-quantitative RT-PCR analysis revealed abnormal pre-mRNA splicing of the following four genes in the *rid1-2/+* and *gfa1-1/+* mutants: *EMBRYO SAC DEVELOPMENT ARREST 26* (AT2G01730), *FOLYLPOLYGLUTAMATE SYNTHETASE 3* (AT3G55630), *AMINOACYL-tRNA LIGASE* (AT1G09620), and *GLYCINE-RICH RNA-BINDING PROTEIN 8* (AT4G39260) (Fig. 8).

**Table 3.** The genes identified in gfa1-1/+ and rid1-2/+ mutants showing abnormal pre-mRNA splicing by qRT-PCR analysis

**Gene ID**	**Protein**	**Function**	**References**
At1g02840	SERINE/ARGININE-RICH PROTEIN SPLICING FACTOR 34	Alternative splicing	Barta *et al.*, 2010
At3g53500	ARGININE/SERINE-RICH ZINC KNUCKLE-CONTAINING PROTEIN 32	Alternative splicing	Barta *et al.*, 2010
At4g02430	SERINE/ARGININE-RICH PROTEIN SPLICING FACTOR 34B	Alternative splicing	Barta *et al.*, 2010
At4g25500	ARGININE/SERINE-RICH SPLICING FACTOR 40	Alternative splicing	Barta *et al.*, 2010
At4g39260	GLYCINE-RICH RNA-BINDING PROTEIN 8; CIRCADIAN RHYTHM, AND RNA BINDING 1	Alternative splicing	Schoning *et al.*, 2008
At1g09620	Leucine-tRNA ligases, aminoacyl-tRNA ligases	FG development	Berg *et al.*, 2005
At2g01730	EMBRYO SAC DEVELOPMENT ARREST 26	FG development	Pagnussat *et al.*, 2005
At2g48140	EMBRYO SAC DEVELOPMENT ARREST 4	FG development	Pagnussat *et al.*, 2005
At3g55630	FOLYLPOLYGLUTAMATE SYNTHETASE ISOFORM 3	Embryo development	Mehrshahi *et al.*, 2010
At5g15230	GAST1 PROTEIN HOMOLOG 4	Reproduction development	Roxrud *et al.*, 2007; Rubinovich and Weiss, 2010
At5g60390	GTP binding Elongation Factor Tu family protein	Translational elongation	Axelos *et al.*, 1989

We also identified several genes that exhibited abnormal splicing in either *rid1-2/+* or *gfa1-1/+* mutants (Supplementary Fig. S6). For example, the first intron retention rate of *CYTOKININ-INDEPENDENT 1* (AT2G47430) and a gene encoding the F-box domain-containing protein (AT2G40910) was higher in the *rid1-2*/+ mutant than in the wild-type or *gfa1-1*/+ mutant plants. In contrast, the first intron retention rate of *EMBRYO SAC DEVELOPMENT ARREST 32* (AT3G62210) and *NOTCHLESS* (AT5G52820) was obviously higher in the *gfa1-1/+* mutant and lower in the *rid1-2*/+ mutant, compared with the wild-type retention rate. Of these genes, three (AT2G47430, AT3G62210, and AT5G52820) are known to regulate FG development in Arabidopsis (Pagnussat *et al.*, 2005; Chantha *et al.*, 2010; Deng *et al.*, 2010). Therefore, there are some differences in the genes required for RID1- or GFA1-mediated splicing during FG development.

We also analyzed the expression of genes involved in FG development, and there were no changes to pre-mRNA splicing in the *gfa1-1/+* and *rid1-2/+* mutants. The expression levels of 30 genes were lower in the ovules of *gfa1-1/+* and *rid1-2/+* mutants than in the wild-type ovules (Supplementary Fig. S7 and Table S3). This result suggested that RID1 and GFA1 function by mediating the expression of genes involved in FG development and pre-mRNA splicing.

## Discussion

The Arabidopsis genome encodes at least 20 DEAH/RHA helicases. A few of these have been identified (e.g. EPS3, AtFAS4, ABO6, and RID1), with activities influencing plant development and abiotic stress tolerance (Tzafrir *et al.*, 2004; Herr *et al.*, 2006; Pogorelko *et al.*, 2008; He *et al.*, 2012; Ohtani *et al.*, 2013; Tsugeki *et al.*, 2015; Howles *et al.*, 2016). However, plant DEAH/RHA helicase functions have not been fully characterized. Our results presented here revealed that recombinant RID1 could unwind dsRNA *in vitro*, with a preferred 3′ to 5′ direction. Yeast PRP22 also exhibits RNA helicase activity by binding single-stranded RNA and unwinding in the 3′ to 5′ direction (Schwer and Meszaros, 2000; Tanaka and Schwer, 2005). The requirement for a single-stranded region in the substrate of DEAH-box helicases may be determined by the conserved domains at the C-terminus of the helicases (Cordin *et al.*, 2012). RID1 can also unwind dsDNA, but exhibits greater RNA helicase activity. In fact, many helicases can function as RNA and DNA helicases. For example, pea DNA helicase 45, which is the first DEAD-box family protein identified with helicase activity in plants, can unwind dsDNA and dsRNA (Pham *et al.*, 2000). Additionally, the viral helicase nucleoside triphosphate phosphohydrolase II has robust RNA helicase activity, with relatively minor effects on DNA substrates. Interestingly, this helicase can catalyze reactions involving DNA–RNA hybrid double strands (Taylor *et al.*, 2010). Whether other Arabidopsis DEAH-box helicases have DNA and RNA helicase activities remain to be determined.

Previous studies reported that the *rid1* mutants were defective in cell specification of mature FGs (Ohtani *et al.*, 2013). In this study, we determined that the *rid1* mutation led to defective FG development. We also observed abnormal cell specification in mature *rid1* FGs, which was consistent with the findings of Ohtani *et al.* (2013). This result may have been due to delayed development of the mutant FG. Because the FG defects in the *rid1* mutants are similar to those of the *gfa1* mutants (Coury *et al.*, 2007; Moll *et al.*, 2008; Liu *et al.*, 2009), and *RID1* and *GFA1* are similarly expressed, it is likely that RID1 and GFA1 interact with each other. Our results confirmed the interaction between RID1 and GFA1. In addition, mutated RID1 exhibited a decreased ability to interact with GFA1, and could not complement the seed-abortion phenotype of the *rid1-2/+* mutant line. Furthermore, the *rid1* and *gfa1* mutants exhibited similar abnormalities in pre-mRNA splicing of genes required for FG development. These results indicate that the interaction between RID1 and GFA1 is critical for FG development in Arabidopsis.

The RNA helicases are involved in all aspects of RNA metabolism, including gene transcription, pre-mRNA splicing, and mRNA translation (Fairman-Williams *et al.*, 2010). During pre-mRNA splicing in humans and yeast, spliceosomes consist of five snRNPs (i.e. U1, U2, U4, U5, and U6), which function in a stepwise manner (Will and Lührmann, 2011). The U5 snRNP forms the key structure of the spliceosome, and is incorporated into the U4/U6.U5 tri-snRNP. PRP8, BRR2, and SNU114/U5-116kD participate in the formation of U5 snRNP complexes in humans and yeast. In Arabidopsis, *GFA1*, *AtBRR2a*, and *AtPRP8a*, the homologs of yeast *SNU114*, *BRR2*, and *PRP8*, respectively, are similarly expressed during Arabidopsis development (Supplementary Fig. S5). RID1 may influence snRNP biogenesis and consequently spliceosome assembly (Ohtani *et al.*, 2013). These findings suggest that RID1 interacts with the U5 snRNP complex in Arabidopsis. In yeast (*S. cerevisiae*), seven DEAH/RHA helicases have been identified (i.e. DHR1, DHR2, YL419W, PRP2, PRP16, PRP22, and PRP43). Of these, PRP2, PRP16, PRP22, and PRP43 are involved in yeast pre-mRNA splicing through cooperative activities with the snRNP spliceosome (Will and Lührmann, 2011; Hoskins and Moore, 2012). However, there has so far been no evidence that these four DEAH/RHA helicases directly interact with SNU114 during pre-mRNA splicing. Moreover, RID1 could not rescue the cold-sensitive growth of the yeast *prp22* mutant. Additionally, substitutions of single RID1 amino acids influenced not only intron removal, but also the recognition of the splicing site (Ohtani *et al.*, 2013). These observations suggest that there are differences in the activities of RID1 and PRP22 during pre-mRNA splicing. Therefore, unlike in yeast, plant DEAH/RHA helicases may function in pre-mRNA splicing by directly interacting with the U5 snRNP complex.

Genetic analyses have shown that yeast SNU114 interacts with proteins, including PRP8, BRR2, PRP28, PRP19, SAD1, and SNU66 (Brenner and Guthrie, 2005). PRP28 is a DEAD-box helicase required for the substitution of U1 snRNP for U6 snRNP. BRR2 is a DEIH-box helicase. The GTP-bound Snu114 activates the RNA helicase BRR2, which mediates the release of U4 snRNP from U6 snRNP during spliceosome activation, and the release of U2 snRNP from U6 snRNP during spliceosome disassembly (Small *et al.*, 2006). Spliceosome activation and disassembly regulated by SNU114/U5-116kD in yeast and humans is commonly induced by conformational changes to the spliceosome catalyzed by BRR2 (Frazer *et al.*, 2008). Therefore, RID1 interacts with GFA1 during the regulation of FG development, and may function in spliceosome activation and disassembly for pre-mRNA splicing.

## Supplementary data

Supplementary data are available at *JXB* online.

Table S1. Primers used in this study.

Table S2. Analysis of the genetic transmission of *rid1* alleles.

Table S3. Down-regulated genes involving in FG development in *rid1*-2/+ and *gfa1*-1/+ mutant ovules identified by qRT-PCR analysis.

Figure S1. T-DNA insertion mutant lines of *RID1* and their phenotypes.

Figure S2. The stages of FG development of wild-type Arabidopsis.

Figure S3. Phylogenetic analysis of RID1 with its orthologs of eukaryotic proteins.

Figure S4. Sequence alignment of RID1 and its orthologs.

Figure S5. The similar expression patterns among *RID1*, *GFA1*, *AtPRP8*, and *AtBRR2a* during Arabidopsis development.

Figure S6. Genes showing abnormal pre-mRNA splicing in either *gfa1-1*/+ or *rid 1-2*/+ mutants.

Figure S7. Genes showing reduced expression in *gfa1-1*/+ and *rid1-2*/+ mutants.

Supplementary Data
