# Supplementary material for: Interaction between RNA helicase ROOT INITIATION DEFECTIVE 1 and GAMETOPHYTIC FACTOR 1 is involved in female gametophyte development in Arabidopsis
Source: J Exp Bot. 2016 Sep 28;67(19):5757–68. doi: 10.1093/jxb/erw341 (PMC5066494; doi:10.1093/jxb/erw341)
Supplement: Supplementary Data [file supp_67_19_5757__index.html]

Interaction between RNA helicase ROOT INITIATION DEFECTIVE 1 and GAMETOPHYTIC FACTOR 1 is involved in female gametophyte development in Arabidopsis — Interaction between RNA helicase ROOT INITIATION DEFECTIVE 1 and GAMETOPHYTIC FACTOR 1 is involved in female gametophyte development in Arabidopsis — Supplementary Data 

# Interaction between RNA helicase ROOT INITIATION DEFECTIVE 1 and GAMETOPHYTIC FACTOR 1 is involved in female gametophyte development in Arabidopsis

## Supplementary Data

Data files

- Supplementary\_Tables\_S1\_S3\_Supplementary\_Figures\_S1\_S7.pdf - Supplementary Data
