# Supplementary material for: Interaction between RNA helicase ROOT INITIATION DEFECTIVE 1 and GAMETOPHYTIC FACTOR 1 is involved in female gametophyte development in Arabidopsis
Source: J Exp Bot. 2016 Sep 28;67(19):5757–68. doi: 10.1093/jxb/erw341 (PMC5066494; doi:10.1093/jxb/erw341)
Supplement: Supplementary Data [file supp_erw341_Supplementary_Tables_S1_S3_Supplementary_Figures_S1_S7.pdf]

Supplementary data

Table S1. Primers used in this study.

**Primers**	**sequence**
RID1LP1	5'-AGCACCCAATCAGGGATATTC-3'
RID1RP1	5'-ACAGACAACAACAAGGGATGC-3'
RID1LP2	5'-AGAATATCCCTGATTGGGTGC-3'
RID1RP2	5'-AACAGAAGTTGGGTGGATGTG-3'
T-DNALB	5'-ATATTGACCATCATACTCATTGC-3'
RID1-GC-UP	5'-GGCTCGAGCAAGCGGTGAGACAGACGTGGAT-3'
RID1-GC-DOWN	5'-AAGGTACCTCACTCTGCCGTTTGGAAATGGTGAG-3'
RID1-IF	5'-GAATTGATTTAGGTGACACTATAGATCCGCGTGAGAAGAGAGAA-3'
RID1-IR	5'-GAATTGTAATACGACTCACTATAGGGTTCTTGCTGGTTTGCATG-3'
GFA1-GST-UP	5'-TTGGATCCATGGAAAGTAGCTTGTATGATGAG-3'
GFA1-GST-DOWN	5'-TTCTCGAGAATCATCTGCAGATGGAGATCGCCT-3'
RID1-HIS-UP	5'-TAGAATTCATGCCATCAATGGCGCAAGGAGAACTCAAG-3'
RID1-HIS-DOWN	5'-AACTCGAGTCACTCTGCCGTTTGGAAATGGTGAGGAGC-3'
BIFC-RID1N-UP	5'-AAACTAGTATGCCATCAATGGCGCAAGGAG-3'
BIFC-RID1N-DOWN	5'-TTGTCGACCTCCTTGCGCCATTGATGGCAT-3'
BIFC-GFA1C-UP	5'-TTGGATCCATGGAAAGTAGCTTGTATGATGAG-3'
BIFC-GFA1C-DOWN	5'-TTCTCGAGAATCATCTGCAGATGGAGATCGCCT-3'

Yeast two-hybrid

RID1-AD-UP	5'-TAGAATTCATGCCATCAATGGCGCAAGGAGAACTCAAG-3'
RID1-AD-DOWN	5'-AACTCGAGTCACTCTGCCGTTTGGAAATGGTGAGGAGC-3'
RID1-1-309-UP	5'-AAGAATTCATGCCATCAATGGCGCAAGGA-3'
RID1-1-309-DOWN	5'-TTTCTCGAGTCTCTCAACAGATTCAATTTC-3'
RID1-265-472-UP	5'-AAGAATTCCTTTACACTGTTCATCCTGAA-3'
RID1-265-472-DOWN	5'-TTGGATCCTATAATCGCCCCCCTTGAAGG-3'
RID1-453-612-UP	5'-TTGAATTCATTGATGATATAGTTGGATTT-3'
RID1-453-612-DOWN	5'-AAGGATCCCCTATATATGTCACGAGCATG-3'
RID1-564-717-UP	5'-TTGAATTCAGTGTTTATCGGGAGTCAGAT-3'
RID1-564-717-DOWN	5'-AAGGATCCCTCTGCCGTTTGGAAATGGTG-3'
RID1-1-271-UP	5'-AAGAATTCATGCCATCAATGGCGCAAGGAGA-3'
RID1-1-271-DOWN	5'-TTGAGCTCTTCAGGATGAACAGTGTAAAGAAT-3'
RID1-272-460-UP	5'-AAGAATTCTCAGATTATGTAGATGCCACCT-3'
RID1-272-460-DOWN	5'-TTGAGCTCAAATCCAACTATATCATCAATCCC-3'
RID1-1-265-UP	5'-AAGAATTCATGCCATCAATGGCGCAAGGAGA-3'
RID1-1-265-DOWN	5'-TTGAGCTCAAGAATGTCAACAGGAAATTGTCGCC-3'
RID1-1-256-UP	5'-AAGAATTCATGCCATCAATGGCGCAAGGAGA-3'
RID1-1-256-DOWN	5'-TTGAGCTCCTGCACATGAACAGCTTTGGCA-3'
RID1-1-181-UP	5'-AAGAATTCATGCCATCAATGGCGCAAGGAGA-3'
RID1 1-181-DOWN	5'-TTGAGCTCTATTTTTTTCAGCAAAGCCAGCAG-3'
RID1 1-271 M^{267}-UP	5'-AAGAATTCATGCCATCAATGGCGCAAGGA-3'
RID1 1-271 M^{267}-DOWN	5'-TTGAGCTCTTCAGGATGAACAATGTAAAGAAT-3'
RID1 1-271 $M^{266, 267}$-UP	5'-AAGAATTCATGCCATCAATGGCGCAAGGA-3'

RID1 1-271 $M^{266, 267}$-DOWN	5'-TTGAGCTCTTCAGGATGAACAATGAAAAGAA-3'
RID1 1-271 $M^{270, 271}$-UP	5'-AAGAATTCATGCCATCAATGGCGCAAGGA-3'
RID1 1-271 $M^{270, 271}$-DOWN	5'-TTGAGCTCTTTAGAATGAACAGTGTAAAGAA-3'
RID1 1-271 $M^{257, 262, 266}$-UP	5'-AAGGACATTCTTGCCACTGTTCATC-3'
RID1 1-271 $M^{257, 262, 266}$-DOWN	5'-AGGAAATTGTCGCGCCTGCACATGA-3'
RID1 1-271 $M^{266, 270, 271}$-UP	5'-AAGAATTCATGCCATCAATGGCGCAAGGAGA-3'
RID1 1-271 $M^{266, 270, 271}$-DOWN	5'-TTGAGCTCTGCTTTATGAACAGCGGCAAGAAT-3'
RID1 1-271 $M^{257-271}$-UP	5'-AAGAAGGACAAACTTGCCGCTGTTC-3'
RID1 1-271 $M^{257-271}$-DOWN	5'-AAATTGTGCCGCCTGCACATGAACA-3'
Semi-quantitative RT-PCR	
qTUB2-LP	5'-GAATGCTGATGAGTGCATGG-3'
qTUB2-RP	5'-AGAAGTGAAGACGGGGGAAT-3'
qAT2G01730-LP	5'-GCCCCCTTCAAAATTTTCTC-3'
qAT2G01730-RP	5'-TTGTGAAACTTTGAAGAAGCAAA-3'
qAT3G55630-LP	5'-TGAAACTTGGGAAATTTTTGG-3'
qAT3G55630-RP	5'-CCGGCAAACAAACAGAAAAC-3'
qAT1G09620-LP	5'-CCCACTGGCTCTGTCTCTGT-3'
qAT1G09620-RP	5'-TCTACCAGCTTGGGGAGAGA-3'
qAT4G39260-LP	5'-TTGAGTACCGGTGCTTTGTC-3'
qAT4G39260-RP	5'-CCGTCGAGGATCAGAATCAT-3'
**qRT-PCR**	

AT2G47430-LP	5'-GTTTCGACTTTGCTCCGAGG-3'
AT2G47430-RP	5'-CAGGACGCAAAAGACCACAA-3'
AT2G40910-LP	5'-ACGCTTCCTTTATGCAATCTCC-3'
AT2G40910-RP	5'-GAGACAACAAAACAGCAATTGGA-3'
AT3G62210-LP	5'-ACACATCGACACACACACAA-3'
AT3G62210-RP	5'-TTGACTACCTCGATCGTTGTT-3'
AT5G52820-LP	5'-TTGCAGAAACATGACGCCTC-3'
AT5G52820-RP	5'-CACCACAACCACACTGCTTA-3'
AT1G01960-LP	5'-TGTTCCATAATGAGTTGATGCCA-3'
AT1G01960-RP	5'-TGCTTGATAGTTGCTTGGACAG-3'
AT3G06400-LP	5'-AATGCAATGGATTCGGCGTT-3'
AT3G06400-RP	5'-ACCATGCGCAGACATTCAAT-3'
AT3G03810-LP	5'-ATACACCCTCCCTAATGGCC-3'
AT3G03810-RP	5'-TCGTTGCTGCCTAAATGCTC-3'
AT2G35940-LP	5'-TCAACCAACAAAGTAACGTGAG-3'
AT2G35940-RP	5'-TTTTGCTCTTCCGATTTCTCAAA-3'
AT2G34920-LP	5'-ACCTGTGCAACAAGGAATAGTG-3'
AT2G34920-RP	5'-CTAGCAAACGCCCCAAACAT-3'
AT2G34860-LP	5'-CATTCTTGTTGGTTCTGTGTGT-3'
AT2G34860-RP	5'-AATTAGAAACCTTTTGCAGCTGA-3'
AT2G20490-LP	5'-TCGGACACATTGAGTTTGGT-3'
AT2G20490-RP	5'-GTGTCAAAACCAAGCGCAAT-3'
AT4G00310-LP	5'-CAGCATTATCCTCCGCCTCT-3'
AT4G00310-RP	5'-ACGACCCATTAACCCTAATCGA-3'
AT4G00020-LP	5'-TAACAACCAAACTCAAGAACCTG-3'
AT4G00020-RP	5'-GCTGTGTGCGCGTTTACATA-3'
AT3G60360-LP	5'-ACTCGAGCTCATCAACTTAACG-3'
AT3G60360-RP	5'-TTCTTTCGTTTCGTCTGTTCG-3'
AT3G56990-LP	5'-ACGAGCCTTTAAGAGACAGGG-3'

AT3G56990-RP	5'-CCAGGTAAGTTTCACCTCTTGT-3'
AT3G59550-LP	5'-TGGGCATTCATCAAATTCCGA-3'
AT3G59550-RP	5'-TGCAAAATTCTCGAAAAGTCAGA-3'
AT3G63130-LP	5'-TGCAGTGTGATTTTGAGGGT-3'
AT3G63130-RP	5'-TCGAAAACGAAGATGGTGTCG-3'
AT4G00140-LP	5'-TTGCCTTCAAGCCAGAGAGT-3'
AT4G00140-RP	5'-GGTGATGGTGATGAGGACGA-3'
AT3G12280-LP	5'-GAACCATGCCCAACCCATTC-3'
AT3G12280-RP	5'-CCGGAGTGGTGGGTTTTAGA-3'
AT1G59680-LP	5'-TCTTTCACTCCGACGGCTTA-3'
AT1G59680-RP	5'-TGTTTTCTGGCTCGAATCCA-3'
AT1G78770-LP	5'-TTTCACTTGTTTTGATTGGGACT-3'
AT1G78770-RP	5'-TTACCGATAAACCCTAAAACCCT-3'
AT1G72970-LP	5'-TGGTCGTTGATGATTCTGTCT-3'
AT1G72970-RP	5'-GGACTGTGCACTAGTGTCCT-3'
AT1G72440-LP	5'-GAAAACTACGGACCATGCCG-3'
AT1G72440-RP	5'-CCTATCACCACTCTGCTCGT-3'
AT4G02060-LP	5'-TCTCTCTTCTTCTCCGTTCTCG-3'
AT4G02060-RP	5'-GGGAGATTCGCGAAAACCC-3'
AT5G66760-LP	5'-CTCTCTTCCTTCTCCTTCCGT-3'
AT5G66760-RP	5'-TTTGCTCGACTACTAACGCA-3'
AT5G52460-LP	5'-TGTGATCAAACCTAACAGCCA-3'
AT5G52460-RP	5'-ACTTTGTGCATCCAACTCGG-3'
AT5G48030-LP	5'-CTCTCAATTGGTTTCGAATTGGT-3'
AT5G48030-RP	5'-AAACTCAATCACACACAGAACAA-3'
AT4G14040-LP	5'-GCAAAAGCTCTCAAAACATTACC-3'
AT4G14040-RP	5'-AGTCCCTTGGCAATAGTTGG-3'
AT4G14220-LP	5'-AAGCTCTTCGGGGTTTAACG-3'
AT4G14220-RP	5'-CGCAAGGCACAAAATCCGAT-3'

AT4G14790-LP	5'-GCAACACGAAGCCATCATAAG-3'
AT4G14790-RP	5'-GCAGCTTCTACATCTGTTTCTGT-3'
AT4G30930-LP	5'-TCTCACTCTCACTGATGCTGA-3'
AT4G30930-RP	5'-CACAAATGCAAAGGTTCGTCC-3'
AT5G05920-LP	5'-CTTAACCCAGTGCCCCAAAC-3'
AT5G05920-RP	5'-GCTTTTCCAATTCAGCAACGG-3'
AT5G19320-LP	5'-AGGAAGGAACATCGGTGCTT-3'
AT5G19320-RP	5'-TCCGTTTATTCTCATCGCAAAC-3'
AT5G22000-LP	5'-GCCTTGATTTTGGGGACTCG-3'
AT5G22000-RP	5'-CTCTCCCGCTCCCTAACAAA-3'
AT2G01730-LP	5'-TCAGCTCAACAAAGTCGTGAC-3'
AT2G01730-RP	5'-CAAATTTATCTTCAGGGACAGCA-3'
AT3G55630-LP	5'-TGACTTTTTCTGACTTTGTGTGTTC-3'
AT3G55630-RP	5'-CTCGTAAATTTTGGCACGACAC-3'
AT1G09620-LP	5'-AATTCTCTCCCCAAGCTGGT-3'
AT1G09620-RP	5'-GACAGTGCCTACCCCAAAAA-3'
AT4G39260-LP	5'-CCCATCGGTACGTTCATCTT-3'
AT4G39260-RP	5'-TCAGCAAAAACAATCGTGGA-3'
TUB2-LP	5'-TGGCATCAACTTTCATTGGA-3'
TUB2-RP	5'-ATGTTGCTCTCCGCTTCTGT-3'

Table S2. Analysis of the genetic transmission of *rid1* alleles[§]

Genotype	***RID1*/-**	***RID1*/+**	**TE (%)**
rid1-2/+ ♀ × Wild type ♂	0	123	0*
rid1-2/+ ♀ × Wild type ♂ [¶]	9	162	5.6*

§ The genetic transmission of *RID1* was analyzed by genotyping F1 progeny of the denoted crosses. The transmission efficient (TE) = (*rid1*/+) / (*RID1*/+) × 100% was calculated according to Howden et al. (1998).

¶ The pistils of *rid1-2*/+ plants at floral stage 12 were pollinated with pollen of wild-type plants at 72 h after emasculation.

* indicating the significant difference between the two of them ($p < 0.01$).

Reference: Howden, R., Park, S.K., Moore, J.M., Orme, J., Grossniklaus, U., et al. (1998) Selection of T-DNA-tagged male and female gametophytic mutants by segregation distortion in *Arabidopsis*. Genetics **149**:621–631.

Table S3. Down-regulated genes involving in FG development in *rid1*-2/+ and *gfa1*-1/+ mutant ovules identified by qRT-PCR analysis.

Gene ID	Protein	Function	Reference
At1g01960	EMBRYO SAC DEVELOPMENT ARREST10	FG* development	Pagnussat et al., 2005
At1g59680	EMBRYO SAC DEVELOPMENT ARREST1	FG development	Pagnussat et al., 2005
At1g72440	SLOW WALKER2	FG development and cell cycle progression	Pagnussat et al., 2005; Li et al., 2009
At1g72970	EMBRYO SAC DEVELOPMENT ARREST17	FG development	Pagnussat et al., 2005
At1g78770	ANAPHASE PROMOTING COMPLEX 6	FG development and cell cycle progression	Kwee et al., 2003
At2g20490	EMBRYO SAC DEVELOPMENT ARREST27	FG development.	Pagnussat et al., 2005
At2g34860	EMBRYO SAC DEVELOPMENT ARREST3	FG development.	Pagnussat et al., 2005
At2g34920	EMBRYO SAC DEVELOPMENT ARREST18	FG and MG# development	Pagnussat et al., 2005; Boavida et al., 2009
At2g35940	BEL-like homeodomain protein 1, EMBRYO SAC DEVELOPMENT ARREST29	FG development	Pagnussat et al., 2007
At3g03810	EMBRYO SAC DEVELOPMENT ARREST30	FG development	Pagnussat et al., 2005
At3g06400	Chromatin remodeling protein 11, SWI2/SNF2	FG development	Huanca-Mamani et al., 2005
At3g12280	RETINOBLASTOMA-RELATED protein RBR1	FG and MG development, and cell cycle progression	Johnston et al., 2008
At3g56990	EMBRYO SAC DEVELOPMENT ARREST7	FG development	Pagnussat et al., 2005
At3g59550	SISTER CHROMATID COHESION 1 PROTEIN 3	FG and MG development	Jiang et al., 2007
At3g60360	EMBRYO SAC DEVELOPMENT ARREST14	FG development	Pagnussat et al., 2005; Jiang et al., 2007
At3g63130	RAN GTPase activating protein 1	FG development and cell cycle	Rodrigo-Peiris et al., 2011

		progression	
At4g00020	EMBRYO SAC DEVELOPMENT ARREST20, BREAST CANCER 2 LIKE 2A	FG development	Pagnussat et al., 2005
At4g00140	EMBRYO SAC DEVELOPMENT ARREST34	Polar nucleus fusion and FG development	Pagnussat et al., 2005
At4g00310	EMBRYO SAC DEVELOPMENT ARREST8	FG development	Pagnussat et al., 2005
At4g02060	PROLIFERA	FG development and cell cycle progression	Springer et al., 2000
At4g14040	SELENIUM-BINDING PROTEIN 2, EMBRYO SAC DEVELOPMENT ARREST38	FG development	Pagnussat et al., 2005
At4g14220	RING-type E3 ubiquitin ligase RHF1A	FG development and cell cycle progression	Liu et al., 2008
At4g14790	EMBRYO SAC DEVELOPMENT ARREST15	FG and MG development.	Pagnussat et al., 2005; Boavida et al., 2009; Gagliardi et al., 1999
At4g30930	NUCLEAR FUSION DEFECTIVE 1	FG and MG development	Portereiko et al., 2006
At5g05920	EMBRYO SAC DEVELOPMENT ARREST22	FG development	Pagnussat et al., 2005
At5g19320	RAN GTPase activating protein 2	FG development and cell cycle progression	Rodrigo-Peiris et al., 2011
At5g22000	RING-type E3 ubiquitin ligase RING-H2 GROUP F2A	FG development and cell cycle progression	Liu et al., 2008
At5g48030	GAMETOPHYTIC FACTOR 2	FG development	Christensen et al., 1998
At5g52460	EMBRYO SAC DEVELOPMENT ARREST41	FG development and cell cycle progression	Pagnussat et al., 2005
At5g66760	SUCCINATE DEHYDROGENASE 1-1	FG and MG development	León et al., 2007

*FG, Female gametophyte; # MG, Male gametophyte.

References:

Boavida, LC., Shuai, B., Yu, HJ., Pagnussat, GC., Sundaresan, V., et al. (2009). A collection of Ds insertional mutants associated with defects in male

gametophyte development and function in *Arabidopsis thaliana*. Genetics **181**:1369–1385.

Christensen, CA., Subramanian, S., and Drews, GN. (1998). Identification of gametophytic mutations affecting female gametophyte development in *Arabidopsis*. Dev. Biol. **202**:136–151.

Gagliardi, D., Kuhn, J., Spadinger, U., Brennicke, A., Leaver, CJ., et al. (1999). An RNA helicase (AtSUV3) is present in *Arabidopsis thaliana* mitochondria. FEBS Lett. **458**:337–342.

Huanca-Mamani, W., Garcia-Aguilar, M., León-Martínez, G., Grossniklaus, U., and Vielle-Calzada, JP. (2005). CHR11, a chromatin-remodeling factor essential for nuclear proliferation during female gametogenesis in *Arabidopsis thaliana*. Proc. Natl. Acad. Sci. USA **102**:17231–17236.

Jiang, L., Xia M., Strittmatter, L.I., and Makaroff, C.A. (2007). The *Arabidopsis* cohesin protein SYN3 localizes to the nucleolus and is essential for gametogenesis. Plant J. **50**:1020–1034.

Johnston, AJ., Matveeva, E., Kirioukhova, O., Grossniklaus, U., and Gruissem, W. (2008). A dynamic reciprocal RBR-PRC2 regulatory circuit controls *Arabidopsis* gametophyte development. Curr. Biol. **18**:1680–1686.

Kwee, H.S., and Sundaresan, V. (2003). The *NOMEGA* gene required for female gametophyte development encodes the putative APC6/CDC16 component of the Anaphase Promoting Complex in *Arabidopsis*. Plant J. **36**:853–866.

León, G., Holuigue, L., and Jordana, X. (2007). Mitochondrial complex II is essential for gametophyte development in *Arabidopsis*. Plant Physiol. **143**:1534–1546.

Li, N., Yuan, L., Liu, N., Shi, D., Li, X., et al. (2009). *SLOW WALKER2*, a NOC1/MAK21 homologue, is essential for coordinated cell cycle progression during female gametophyte development in Arabidopsis. Plant Physiol. **151**:1486–1497.

Liu J., Zhang Y., Qin G., Tsuge T., Sakaguchi N., et al. (2008). Targeted degradation of the cyclin-dependent kinase inhibitor ICK4/KRP6 by RING-type E3 ligases is essential for mitotic cell cycle progression during *Arabidopsis* gametogenesis. Plant Cell **20**:1538–1554.

Pagnussat, G.C., Yu, H.J., Ngo, Q.A., Rajani, S., Mayalagu, S., et al. (2005). Genetic and molecular identification of genes required for female gametophyte development and function in *Arabidopsis*. Development **132**:603–614.

Pagnussat, G.C., Yu H.J., and Sundaresan, V. (2007). Cell-fate switch of synergid to egg cell in *Arabidopsis eostre* mutant embryo sacs arises from misexpression of the BEL1-like homeodomain gene *BLH1*. Plant Cell **19**:3578–3592.

Portereiko, M.F., Sandaklie-Nikolova, L., Lloyd, A., Dever, C.A., Otsuga, D., et al. (2006). NUCLEAR FUSION DEFECTIVE1 encodes the *Arabidopsis* RPL21M protein and is required for karyogamy during female gametophyte development and fertilization. Plant Physiol. **141**:957–965.

Rodrigo-Peiris, T., Xu, X.M., Zhao, Q., Wang, H.J., and Meier, I. (2011). RanGAP is required for post-meiotic mitosis in female gametophyte

development in *Arabidopsis thaliana*. J. Exp. Bot. **62**:2705–2714.

Springer, P.S., Holding, D.R., Groover, A., Yordan, C., and Martienssen, R.A. (2000). The essential Mcm7 protein PROLIFERA is localized to the nucleus of dividing cells during the G(1) phase and is required maternally for early *Arabidopsis* development. Development **127**:1815–1822.

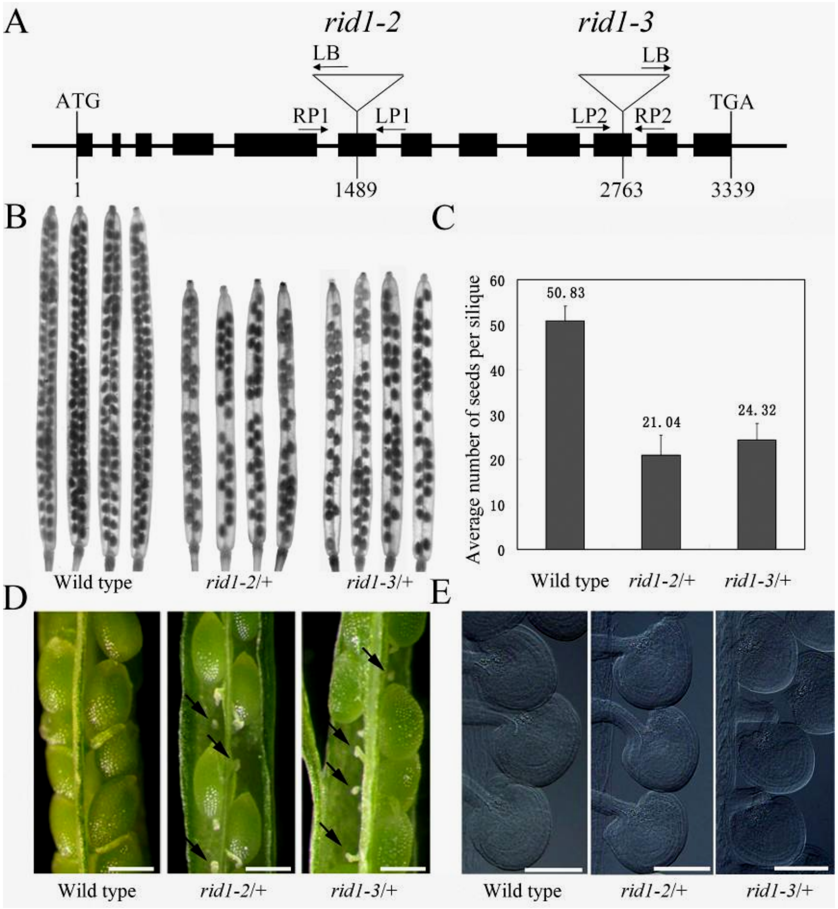

Figure S1. T-DNA insertion mutant lines of *RID1* and their phenotypes.

(A) Two T-DNA insertion lines of *RID1*, *rid1-2* and *rid1-3*. Black boxes indicate the exons of *RID1* genomic sequence, and the lines are introns. The triangles represent the locations of the T-DNA insertion in the mutant lines. LBs primers for left border of the T-DNA, RPs and LPs gene-specific primers at downstream and upstream locations, respectively.

(B) Mature siliques of wild type and *rid1* mutants.

(C) The numbers of seeds in the siliques of *rid1* mutants are about half that of wild type. The data were collected from 150 siliques for each sample.

(D) Parts of the young seeds in *rid1* mutant siliques are abortive (arrows). Bar, 100 μm.

(E) The morphology of the ovules is normal in the mutant lines. Bar, 20 μm.

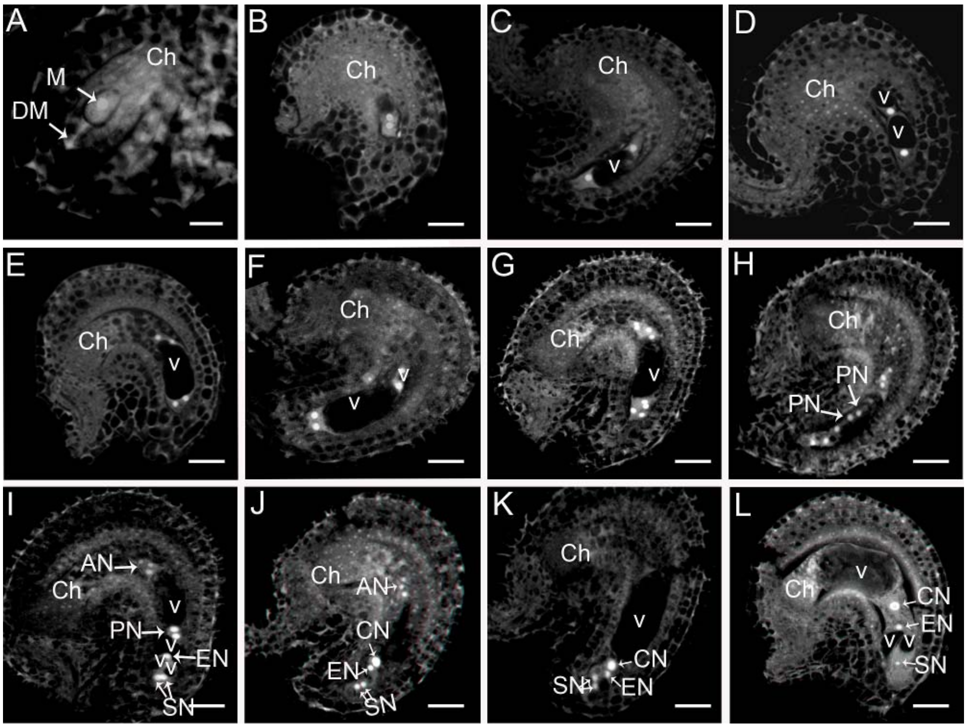

Figure S2. The stages of FG development of wild-type *Arabidopsis*.

FG1 (A), FG2 (B and C), FG3 (D), FG4 (E and F), FG5 (G), FG6 (H, I, and J), FG7 (K), and FG8 (L). AN, antipodal nucleus; Ch, chalazal end; CN, central cell nucleus; DM, degenerated megaspores; DS, degenerated synergid; EN, egg cell nucleus; M, megaspore; PN, polar nucleus; SN, synergid nucleus; V, vacuole. Bars, 10 µm.

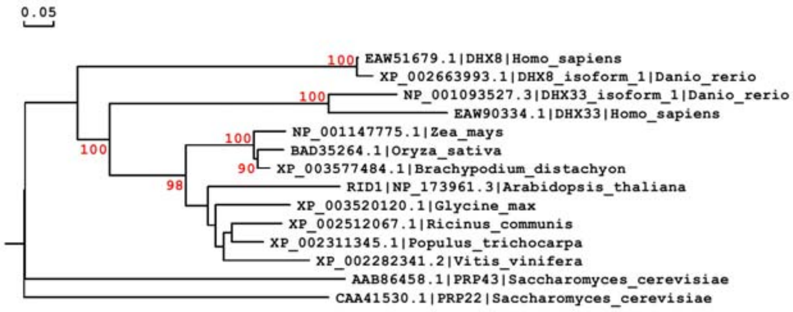

Figure S3. Phylogenetic analysis of RID1 with its orthologs of eukaryotic proteins.

Figure S4

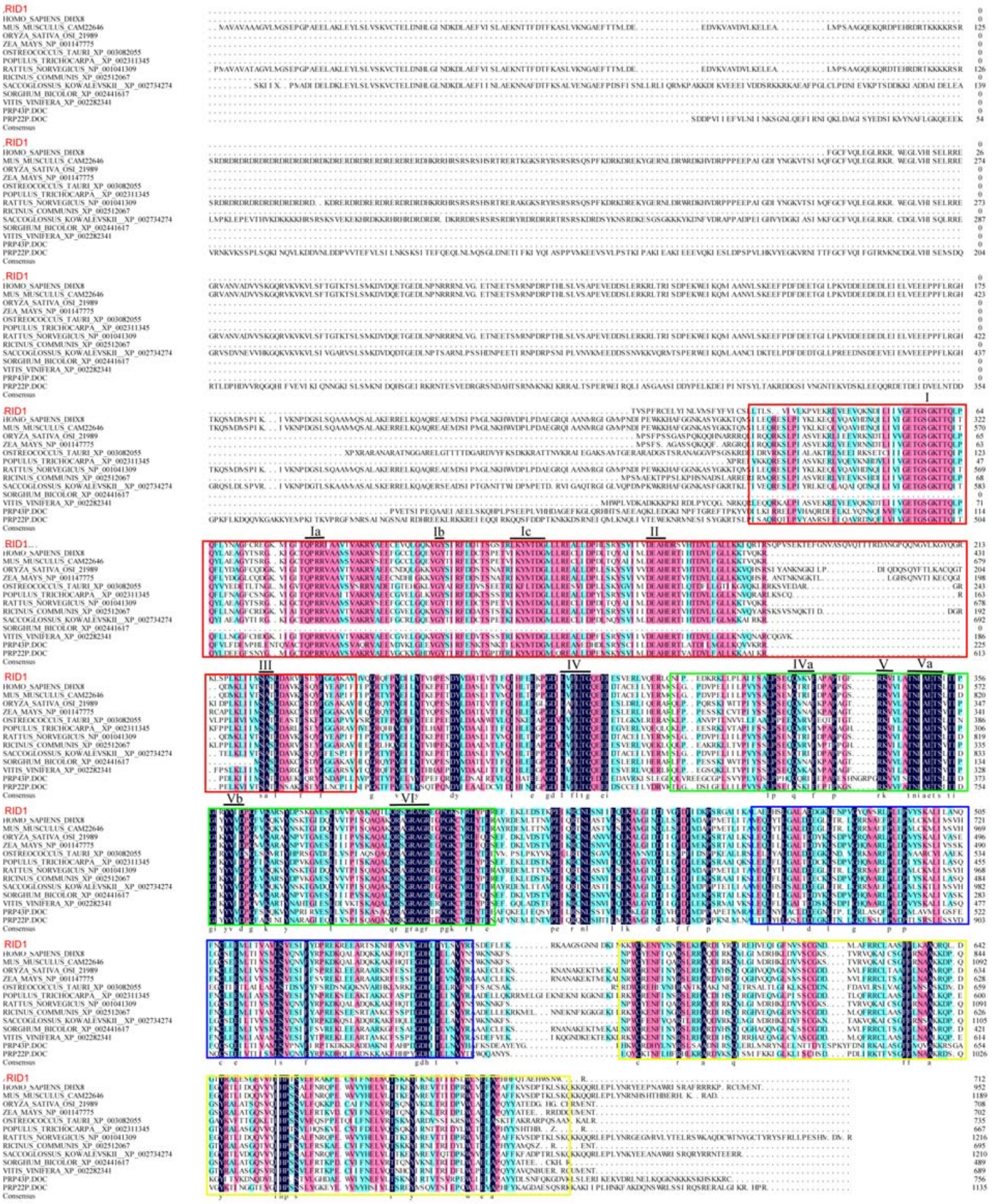

Figure S4. Sequence alignment of RID1 and its orthologs.

The DEXDc, HELICc, HA2, OB-fold domains labeled with red, green, blue, and yellow squares, respectively. Twelve motifs (I, Ia, Ib, Ic, II, III, IV, IVa, V, Va, Vb and VI) in the DEXDc and HELICc domains were labeled in sequence.

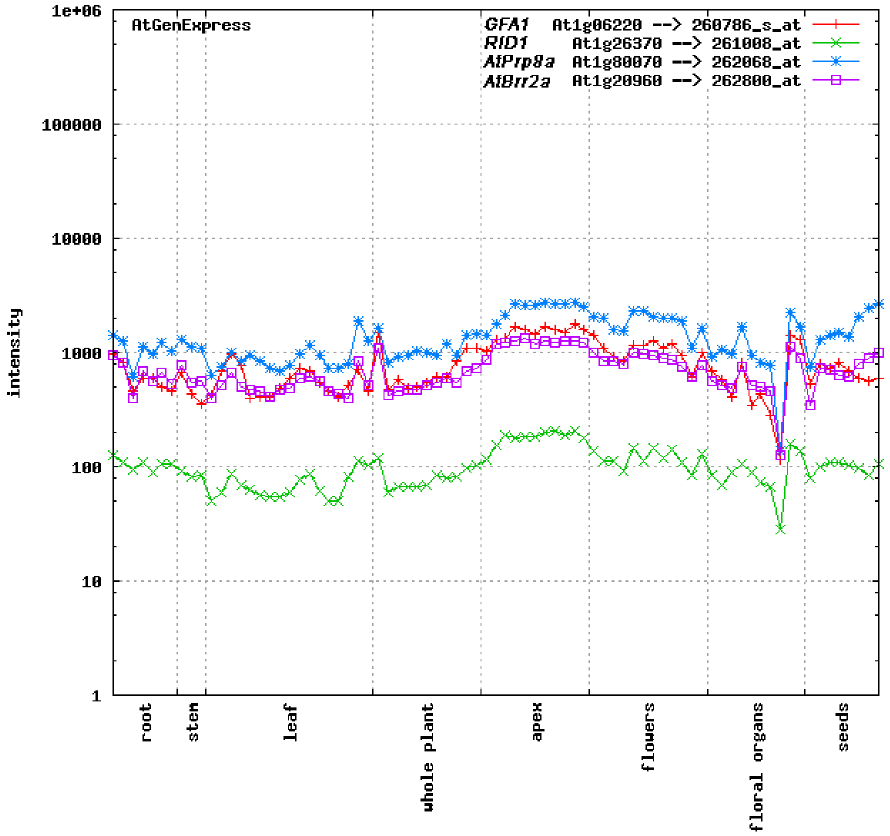

Figure S5. The similar expression patterns among *RID1*, *GFA1*, *AtPRP8*, and *AtBRR2a* during *Arabidopsis* development.

The expression patterns were analyzed by AtGenExpress Visualization Tool (http://jsp.weigelworld.org/expviz/expviz.jsp?).

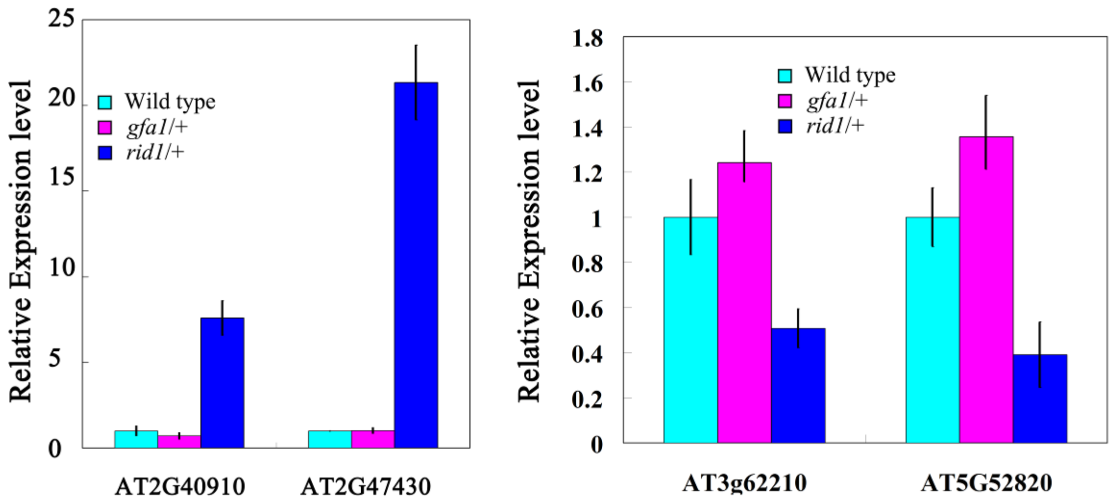

Figure S6. The genes show abnormal pre-mRNA splicing in either *gfa1-1*/+ or *rid 1-2*/+ mutants.

The abnormal retentions of first intron in pre-mRNA of RNA splicing-related and female gametophyte development-related genes in *rid1-2*/+ and *gfa1-1*/+ mutants were detected by qRT-PCR analysis. The expression of *TUBULIN2* was used as the internal control. All experiments were repeated three times.

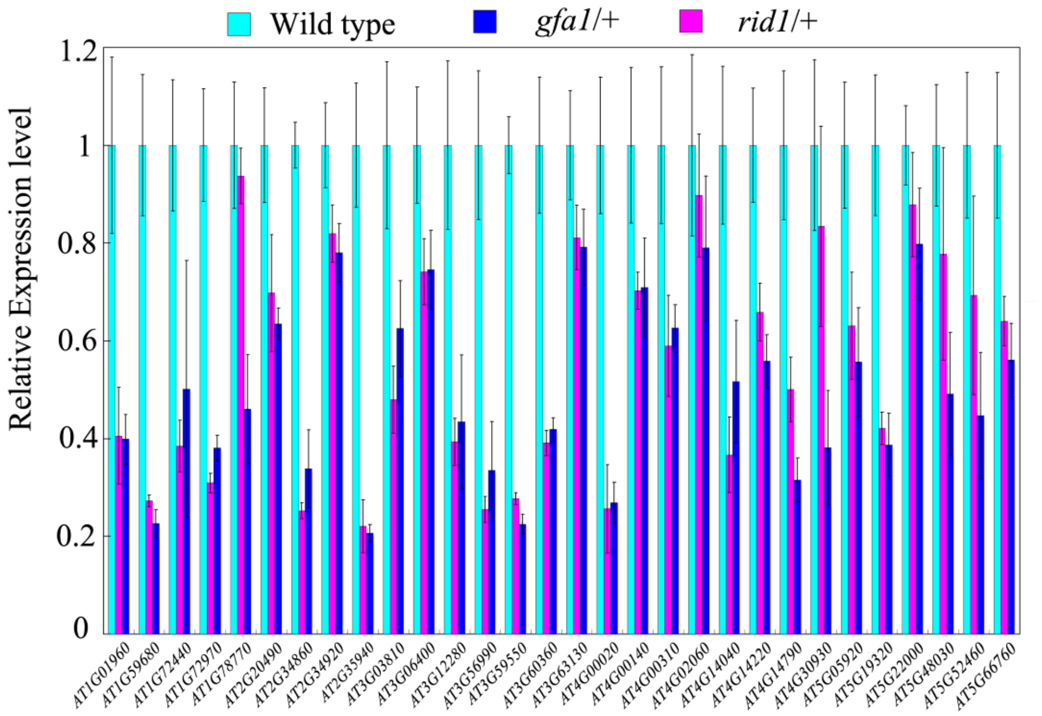

Figure S7. The genes show reduced expression in *gfa1-1*/+ and *rid1-2*/+ mutants. The expressions were detected by qRT-PCR analysis. The expression of *TUBULIN2* was used as the internal control. All experiments were repeated three times.
